# Policy Series: Health and Aging Policy Fellows: Bringing Together Aging and Policy

**DOI:** 10.1093/geroni/igaf122.615

**Published:** 2025-12-31

**Authors:** Maureen Henry, Priti Patel

## Abstract

The Health and Aging Policy Fellows is a program that has trained more than 200 people through a one-year fellowship that includes an intensive training in Washington DC and a placement in Washington, within a state or local government, or with a non-profit organization. Fellows are selected from across career stages, disciplines, and emphasis within aging. Presentations will describe how policy can be used to solve discrete structural challenges that affect older persons, such as unpredictable and non-transparent medication prices or direct care worker shortages. Another presentation will report about processes of collecting and analyzing data to create a national plan for aging. The final presentation will describe aspects of the appropriations process in Congress. Together, presentations will show a breadth of approaches to tackling aging policy challenges.

